# Comparative study between manual injection intraosseous anesthesia and conventional oral anesthesia

**DOI:** 10.4317/medoral.17456

**Published:** 2011-12-06

**Authors:** David Peñarrocha-Oltra, Javier Ata-Ali, María J. Oltra-Moscardó, María Peñarrocha-Diago, Miguel Peñarrocha

**Affiliations:** 1 DDS. Resident of the Master in Oral Surgery and Implantology. Valencia University Medical and Dental School; 2 DDS. Master in Oral Surgery and Medicine. Master in Oral Surgery and Implantology. Valencia University Medical and Dental School; 3 MD. DDS, PhD. Valencia University Medical and Dental School; 4 Associate Professor of Oral Surgery. Valencia University Medical and Dental School; 5 Chairman of Oral Surgery. Director of the Master in Oral Surgery and Implantology. Valencia University Medical and Dental School. Valencia (Spain)

## Abstract

Objective: To compare intraosseous anesthesia (IA) with the conventional oral anesthesia techniques. Materials and methods: A simple-blind, prospective clinical study was carried out. Each patient underwent two anesthetic techniques: conventional (local infiltration and locoregional anesthetic block) and intraosseous, for res-pective dental operations. In order to allow comparison of IA versus conventional anesthesia, the two operations were similar and affected the same two teeth in opposite quadrants. Results: A total of 200 oral anesthetic procedures were carried out in 100 patients. The mean patient age was 28.6±9.92 years. Fifty-five vestibular infiltrations and 45 mandibular blocks were performed. All patients were also subjected to IA. The type of intervention (conservative or endodontic) exerted no significant influence (p=0.58 and p=0.62, respectively). The latency period was 8.52±2.44 minutes for the conventional techniques and 0.89±0.73 minutes for IA – the difference being statistically significant (p<0.05). Regarding patient anesthesia sensation, the infiltrative techniques lasted a maximum of one hour, the inferior alveolar nerve blocks lasted between 1-3 hours, and IA lasted only 2.5 minutes – the differences being statistically significant (p≤0.0000, Φ=0.29). Anesthetic success was recorded in 89% of the conventional procedures and in 78% of the IA. Most patients preferred IA (61%) (p=0.0032). Conclusions: The two anesthetic procedures have been compared for latency, duration of anesthetic effect, anesthetic success rate and patient preference. Intraosseous anesthesia has been shown to be a technique to be taken into account when planning conservative and endodontic treatments.

** Key words:** Anesthesia, intraosseous, oral anesthesia, Stabident®, infiltrative, mandibular block.

## Introduction

Although pain management in dental procedures has progressed in recent decades, dental anesthesia remains a problem for some dentists, mainly as a result of patient fear of the needle and the risk of soft tissue nibbling. Intraosseous anesthesia (IA) allows direct placement of the anesthetic solution in the cancellous bone adjacent to the tooth programmed for anesthesia. Since the anesthetic solution is targeted directly to the tooth requiring treatment, the surrounding soft tissues are usually not affected (1).

In the year 2002 the American Dental Association (ADA) accepted the Stabident® system as an effective and safe technique for intraosseous pulp anesthesia, either as a primary procedure or as a complement to other anesthetic maneuvers. Many studies have been published on this intraosseous anesthesia technique (2-12), with reported successful pulp anesthesia rates of 41-96%, depending on the tooth involved, the pathology, treatment and method used for evaluation (1).

The present study was designed to compare the latency, duration of anesthetic effect, anesthetic success rate and patient preferences in relation to two anesthetic techniques: intraosseous anesthesia and conventional oral anesthesia.

## Material and Methods

A simple-blind, prospective clinical study was carried out. A total of 200 oral anesthetic procedures were carried out in 100 patients. Each patient was subjected to both anesthetic techniques: conventional (local infiltration and locoregional anesthetic block) and intraosseous, for respective dental operations. A 7-day interval was established between the two procedures. Anesthesia in all cases was carried out by the same operator (O-M. MJ). In order to allow comparison of IA versus conventional anesthesia, the two operations were similar and affected the same two teeth in opposite quadrants.

Dental treatment comprised silver amalgam or composite reconstructions and root canal treatments of teeth with vital pulp tissue. In all cases attempts were made to ensure that the treatments on both sides of the mouth were the same.

The following inclusion criteria were established: patients between 10-55 years of age, the absence of disease antecedents (diabetes, heart disease, high blood pressure), the absence of medication, the absence of oral or soft tissue infections, and the confirmation of pulp vitality using thermal and electrical tests. The exclusion criteria were: periodontal (periodontal pockets or tooth mobility) or radiological alterations (bone loss or periapical radiotransparencies), as well as any type of third molar treatment.

All parents gave written informed consent to inclusion in the study, which was carried out according to the Declaration of Helsinki, following approval of the local Ethics Committee.

For conventional anesthesia we used the Aspiject® syringe (Laboratorios Inibsa, Barcelona, Spain) with an auto-aspirating system and a 25-mm injection needle. IA in turn was carried out using the Stabident® system (Fairfax Dental Inc., Miami, FL, USA), following the technique described by Gallatin et al. (4). The anesthetic solution used in the conventional procedures was 2% lidocaine with 1:100,000 adrenalin, while IA was carried out using 3% mepivacaine without vasoconstrictor.

The chi-squared test was used for comparing qualitative variables. The strength of the correlation between categorical variables was evaluated using the phi-square coefficient (Φ). The parametric Student t-test in turn was used to assess differences between the means of two groups. Comparison between the two anesthetic techniques in one same patient was carried out using the McNemar test. Statistical significance was accepted for p≤0.05.

## Results

The mean patient age was 28.6 ± 9.92 years (range 11-55); there were 47 males and 53 females. In relation to the conventional technique, 55 vestibular infiltrations (51 in the upper maxilla, and 4 in lower incisors and canines) and 45 mandibular blocks were carried out. All patients were also subjected to IA. 

The same conservative dental treatments were carried out in both the conventional anesthesia group and in the IA group: silver amalgam or composite reconstructions (79%) and endodontic procedures (21%). No significant differences were observed on evaluating the influence of the type of intervention (conservative or endodontic) with conservative anesthesia and IA (p=0.58 and p=0.62, respectively).

The latency period was 8.52±2.44 minutes (range 2-15) for the conventional techniques and 0.89±0.73 minutes (range 0-4) for IA – the difference being statistically significant (p<0.05).

Regarding the duration of the anesthetic effect, the conventional procedures lasted longer than IA: the infiltrative techniques lasted a maximum of one hour in 69% of the cases (38 patients), the inferior alveolar nerve blocks lasted between 1-3 hours in 84.5% of the cases (38 patients), with a mean of 96.75±55.76 minutes, and IA lasted only 2.5±4.55 minutes – the differences being statistically significant (p≤0.0000, Φ=0.29). In relation to IA it is important to mention that 52% of the patients at no time had the sensation of being anesthetized, and that none of the 100 subjects reported numbness of the soft tissues (lip, cheek or tongue). 

The anesthesia success rate was 89% with the conventional procedures (slight discomfort in 10% and only one patient with mild pain) and 78% with IA (14 patients with mild discomfort, 5 with mild pain and 3 with moderate pain). The differences between the two techniques were statistically significant, but of scant practical relevance (x2=6.836, p=0.034, Φ=0.03). A complete anesthetic effect was recorded with the conventional technique in 98% of the upper teeth and in 80% of the lower teeth, while IA afforded good anesthesia in 86% of the upper teeth and in 69% of the lower teeth. 

Most of the patients (61%) preferred IA, while 21% preferred the conventional technique – the difference being statistically significant (p=0.0032).

## Discussion

According to Coggins et al. (2), the onset of anesthetic action with IA is immediate and affects the targeted tooth and its adjacent mesial and distal counterparts. Leonard (5) evaluated the depth of the anesthetic effect achieved for starting endodontic treatment, applying pressure to the gingival sulcus of the tooth programmed for extraction: with conventional techniques he had to wait 8-17 minutes, while with IA this latency period was a mere 10-120 seconds. These results are similar to those of our own series, where significant differences were observed between the two techniques in terms of the time from injection to the moment in which treatment could be started (8.52 and 0.89 minutes, respectively; p<0.05).

In our study the depth of the anesthetic effect was found to be sufficient, and the patients were able to tolerate the dental treatments – including endodontic procedures in vital teeth. Complete analgesia was recorded in 89% of the conventional procedures – in coincidence with the observations of recent studies (7,13). In comparison, analgesia proved complete in 78% of the IA procedures, in agreement with the observations of other authors who used IA as primary technique in non-inflamed pulp tissue, with success rates of 81-100% (10,14).

As commented by Replogle et al. (12), on considering the success rates and the duration of IA, the true usefulness of the latter is perhaps more as complementary anesthesia than as a primary technique. However, in a study carried out by Jensen et al. (14), the IA success rates were 100% for the first molar and 82-91% for the second premolar, while the corresponding success rates with dental nerve block were 81-83% for the first molar and 73-80% for the second premolar (15,16).

Some authors have studied the combination of inferior dental nerve block with IA, obtaining improved results in terms of anesthesia success. In this context, Dunbar et al. (3), on applying both anesthetic techniques in 40 patients, recorded a 100% anesthesia success rate. This association of the two techniques has been shown to be the best alternative in a number of studies, in application both to anesthetic failure and to teeth with irreversible pulpitis (6,8-11).

In a recent study in children with prior exposure to conventional anesthesia (17), 58.9% claimed to prefer IA. Our own data agree with this observation (61%).

In the present study, the two anesthetic procedures have been compared for latency, duration of anesthetic effect, anesthetic success rate and patient preference. Intraosseous anesthesia has been shown to be a technique to be taken into account when planning conservative and endodontic treatments. In this context, it is possible to work on opposite sides of the mouth in one same visit, thanks to the absence of soft tissue anesthesia.
